# Proteomic Analysis of the Organ of Corti Using Nanoscale Liquid Chromatography Coupled with Tandem Mass Spectrometry

**DOI:** 10.3390/ijms13078171

**Published:** 2012-07-02

**Authors:** Hong Peng, Miao Liu, Jason Pecka, Kirk W. Beisel, Shi-Jian Ding

**Affiliations:** 1 Department of Pathology and Microbiology, University of Nebraska Medical Center, Omaha, NE 68198, USA; E-Mails: hong.peng@unmc.edu (H.P.); mli1@unmc.edu (M.L.); 2 Department of Environmental, Agricultural & Occupational Health, University of Nebraska Medical Center, Omaha, NE 68198, USA; 3 Department of Biomedical Sciences, Creighton University, Omaha, NE 68178, USA; E-Mail: jasonpecka@creighton.edu; 4 Mass Spectrometry and Proteomics Core Facility, University of Nebraska Medical Center, Omaha, NE 68198, USA

**Keywords:** organ of Corti, proteomic analysis, nanoLC-MS/MS

## Abstract

The organ of Corti (OC) in the cochlea plays an essential role in auditory signal transduction in the inner ear. For its minute size and trace amount of proteins, the identification of the molecules in pathophysiologic processes in the bone-encapsulated OC requires both delicate separation and a highly sensitive analytical tool. Previously, we reported the development of a high resolution metal-free nanoscale liquid chromatography system for highly sensitive phosphoproteomic analysis. Here this system was coupled with a LTQ-Orbitrap XL mass spectrometer to investigate the OC proteome from normal hearing FVB/N male mice. A total of 628 proteins were identified from six replicates of single LC-MS/MS analysis, with a false discovery rate of 1% using the decoy database approach by the OMSSA search engine. This is currently the largest proteome dataset for the OC. A total of 11 proteins, including cochlin, myosin VI, and myosin IX, were identified that when defective are associated with hearing impairment or loss. This study demonstrated the effectiveness of our nanoLC-MS/MS platform for sensitive identification of hearing loss-associated proteins from minute amount of tissue samples.

## 1. Introduction

Proteomic approaches for the study of the structure and function of the inner ear have been restricted due to the limited amount of tissue and encasement in a bony labyrinth. Major advancements in understanding the auditory end-organ, the cochlea, were greatly facilitated by using genomic and transcriptomic approaches with major mechanistic insights into the auditory system being associated with the identification of over 150 syndromic and non-syndromic genes that cause hearing defects in humans (http://hereditaryhearingloss.org/) [1–3]. A plethora of spontaneous, induced and genetically-engineered mutant mouse models have also contributed to our understanding of the auditory system [4]. These insights from functional studies of genes involved in hearing loss and deafness are summarized in a number of reviews [5–8]. Approximately 50% of congenital or childhood-onset hearing loss is due to defective genes, with the World Health Organization in 2005 estimating that about 278 million people around the world exhibit moderate to profound hearing impairment [9]. Impairment and loss of hearing tremendously affect people’s life quality. Besides gene-based defects, environmental insults (e.g., noise, infection, and ototoxic drugs) and aging contribute to auditory disease in humans. Hearing loss is the second most frequent debilitation of old age, with approximately 50% of the elderly, 65 years or older, experiencing some degree of hearing loss. This high incidence is likely caused by a culmination of environmental insults producing cumulative cellular injury, genetic predisposition of the individual and accumulated burden of somatic mutations. Loss of the sensory epithelium, namely the auditory hair cells, is a major pathological condition that contributes to sensorineural hearing impairment [10].

**Figure 1:**
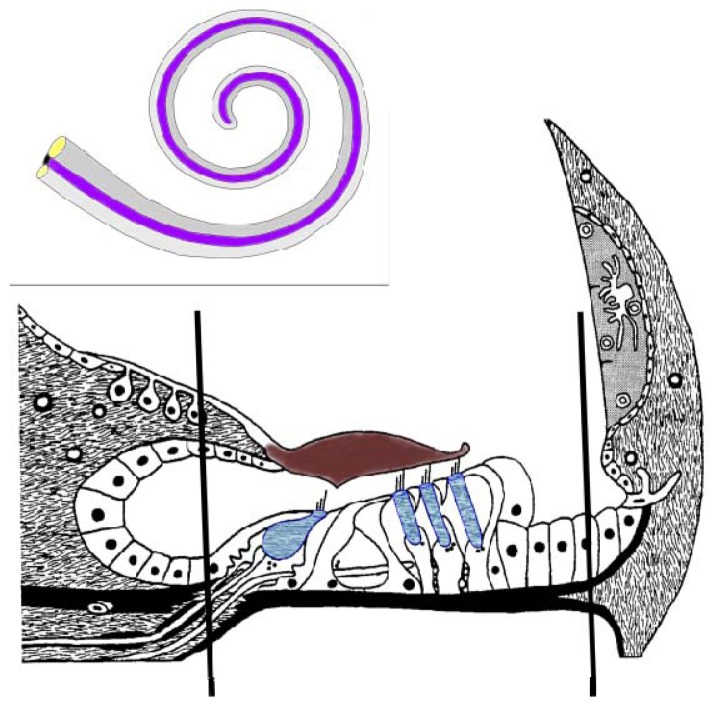
Schematic representation of the cochlear membranous labyrinth and the organ of Corti. A cross section of the cochlea is represented with the hair cells (blue) being depicted with the single inner hair cell and three outer inner hairs. The black lines represent the approximate region isolated by dissection for this proteomic investigation. The framed insert represents the membranous labyrinth of the cochlea with the anterior scala vestibuli (gray), the scala media (cochlear duct) (purple) that contains the organ of Corti and the scala tympani (light gray).

It is now evident that genomic and transcriptomic studies must be complemented with proteomics to reveal dynamic cellular processes *in toto*, since additional complexities can be manifested by post-translational modifications (PTMs), splicing variants, and peptide cleavage products [11]. Furthermore, quantification of protein expression is essential, since it is clearly evident that mRNA concentrations are not necessarily reflected by levels of their corresponding proteins [12,13]. The minimal size, complex morphology and diversified inner ear tissues, fluid compartments containing endolymph and perilymph and bone encapsulated cochlea raise huge limitations on protein profiling of the inner ear [11]. In addition, these limitations have impacted on the ability to follow inner ear development. Another impediment is the absence of adequate human and murine cell lines similar to the developing and adult inner ear that typically facilitate the use of animal models optimal for biological study of the inner ear [14]. Proteomic studies of the inner ear have gained remarkable progress in protein profiling of various inner ear tissues, providing valuable methods to overcome the difficulties of accessing the small number of diverse cell types [15–21]. These samples parallel the diversity of the inner ear tissue composition. Therefore, reduction of the sample complexity by restricting the diversity to the spiral ganglion, the cochlear lateral wall, which includes stria vascularis or the organ of Corti (OC) (Figure 1), has contributed to understanding their tissue specific functions as well as identifications of key molecules in these tissues. Nevertheless, the OC, seated on the basilar membrane, is the sensory epithelium of the cochlear duct, which still reflects a complex tissue. Within the OC a number of different cell types are associated with the inner and outer sulci and there are two histological and functional distinct populations of hair cells (*i.e.*, the inner and outer hair cells) along with their supporting cells, such as the inner pharyngeal, pillar and Deiters cells. There are 16,000–20,000 hair cells in the mammalian OC with a single row of inner hair cells (IHCs) that serve as the primary transmitter of sound signals to the brain and three rows of outer hair cells (OHCs) that function as the cochlear amplifier [22]. A major challenge for the proteomics of the inner ear is to capture the proteomes of these two hair cell types.

Along with the complex heterogeneity of the OC, there is also a unique diversity and composition of expressed genes that are present in the inner ear and this is distinguished by extracellular matrix components, gap junction and adhesion proteins, ion channels and transporters, cell surface proteins and receptors, myosins and other inner ear-associated proteins [18]. Many of these proteins, when altered by mutations, do lead to sensorineural hearing loss in man. For example, myosin-encoding genes, including *MYO1C*, *MYO3A*, *MYO6*, *MYO7A*, and *MYO15A*, play an essential role in hair bundle organization and function, and moreover when mutated, can result in non-syndromic deafness [7]. Although in the last two decades great efforts to identify mutant or target proteins of the inner ear, involved in hearing loss and deafness, were made, a more definite and comprehensive map of the OC proteome will replenish the proteomic dataset of the inner ear and thus facilitate further research in the auditory area.

Recent progress in liquid chromatography coupled with tandem mass spectrometry (LC-MS/MS) based proteomic analysis from small sample sizes has demonstrated the ability to identify thousands of proteins and multiple PTMs simultaneously [23] and now provides an opportunity to examine the OC. Thus, as a high throughput method, LC-MS/MS based proteomics can enrich our limited understanding of the complex proteome of the OC. Besides, insights into the proteome can advance identification of novel signaling molecules and pathways in auditory cell development, differentiation, function, intercellular communication, and disease processes. In our current study we examined normal hearing FVB/N mice to investigate the OC proteome. This permitted the analysis of normal protein expression of the OC, which can be utilized to specify the intricate regulatory processes from genes to proteins, and allows the integration of these data with other “omic” studies. A total of 628 proteins were identified from six replicates of single LC-MS/MS analysis from 10 OCs of 5 normal hearing FVB/N male mice. Although the number of protein identifications is limited, to our knowledge the dataset still represents the largest proteome coverage for the OC reported in the literature so far.

## 2. Results

### 2.1. Identification of Proteins from the OC

**Figure 2:**
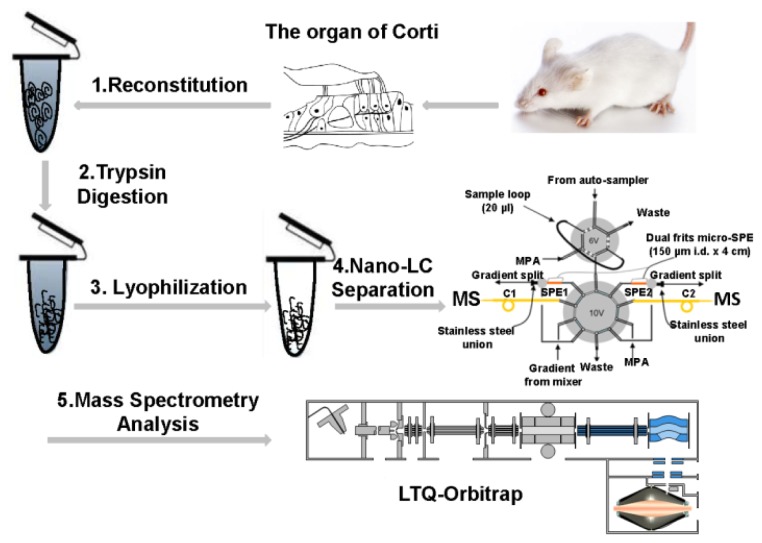
Schematic diagram of proteomic analysis of the mouse organ of Corti (OC) sample. (**1**) The OCs were reconstituted in 100 μL lysis buffer (100 mM ammonium bicarbonate, pH 8.4); (**2**) The lysates were reduced by 5 mM DL-Dithiothreitol (DTT) and digested by trypsin overnight; (**3**) The digests were desalted and dried in a vacuum centrifuge immediately after digestion; (**4**) Dried peptides were subjected to the in-house assembled reverse phase metal-free multiple-column nanoLC system coupled with (**5**) LTQ-Orbitrap XL mass spectrometer for MS analysis.

A nanoLC-MS/MS based proteomic approach was taken to identify proteins from the OC. Figure 2 illustrates the overall strategy applied in this study to investigate the proteome of the OC from FVB/N male mice. OCs were rapidly lysed and underwent trypsin digestion to minimize sample loss and any contamination. Digested peptides were further separated by a nanoLC system and sequenced by a LTQ-Orbitrap XL mass spectrometer. Six LC-MS/MS analyses were introduced into Open Mass Spectrometry Search Algorithm (OMSSA) search engine. A total of 628 proteins with 1074 unique peptides by sequence from 4679 spectra have been identified by OMSSA with false discovery rate (FDR) of 1% (Table S1). Specifically, 175 (27.8%) proteins were mapped with more than one unique peptide. Several proteins identified in the LC-MS/MS analyses have been shown to be involved with hearing impairment (Table 1).

**Figure 3:**
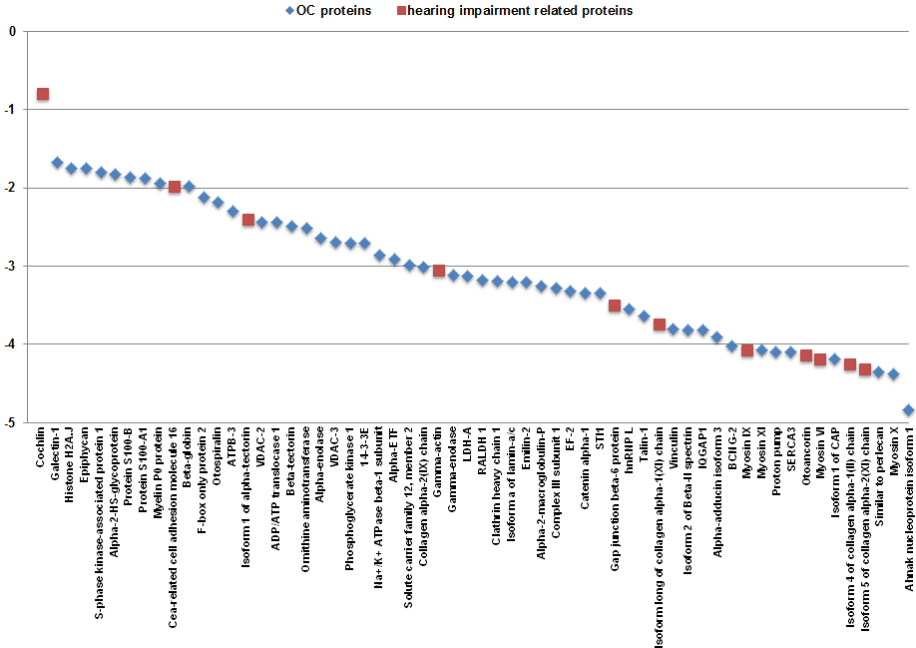
Dynamic range of 60 selected proteins from the mouse OC sample. All log10 values were based on normalized spectra abundance factor (NSAF) values which were used to normalize spectral count. Proteins identified in the OC and the hearing impairment related proteins were labeled as blue diamonds and red squares, respectively.

Semi-quantitative analyses of proteins identified from the OC were determined by normalized spectra abundance factor (NSAF) [3]. NSAF values of identified proteins range from 1.46 × 10^−5^ to 0.16, indicating that the proteome of OC has a dynamic range of 10^4^. Moreover, NSAF values of 99 proteins (15.8% of the total identified proteins) are greater than 0.002 and these comparatively abundant proteins were identified with 2680 MS/MS spectra (64.9% of the total MS/MS spectra). Among all identified proteins, cochlin (COCH) is the most abundant for its identification with 1062 MS/MS spectra (25.7% of the total MS/MS spectra). Moreover, in the proteins of NSAF > 0.002, 60.6% of them are cytoplasmic proteins, including enzymes, transporters, peptidases and ion channels, and 15.2% of them are located in extracellular matrix. Of the eight remaining proteins with NSAF > 0.002, there were two identified as histones and six other proteins, which were ascertained as being plasma membrane associated proteins. Figure 3 plots the log10 NSAF values of 60 selected proteins identified from the OC in this study and clearly shows the distribution of NSAF values. It directly demonstrates 4 orders of magnitude of the proteins identified by our nanoLC-MS/MS platform. Eleven hearing impairment related proteins plotted in the figure were obviously distributed primarily within the low to mid range except COCH. Proteomic studies on these hearing impairment related proteins are critical in unveiling the complexity of auditory disease process, but their low abundance greatly requires the further improvement of the sensitivity of nanoLC-MS/MS analysis.

### 2.2. Identification of PTMs from the OC

Data from the OMSSA search engine identified 174 MS/MS spectra that mapped to 28 modified proteins. Altogether, 13 phosphorylated and 15 acetylated peptides (17 phosphorylated sites and 15 acetylated sites) mapped to 11 and 13 proteins, respectively. Among the phosphopeptides, 7 had a single phosphorylated site and 6 had two phosphorylated sites. In addition, 13 of the identified phosphorylated sites were identified for the first time. Fifteen acetylated sites contained 11 acetylated *N*-termini and 4 acetylated lysines. A phosphopeptide (HAFSPVASVESASGETLHSPK) belonging to α-2-HS-glycoprotein was detected by our MS/MS analysis (Figure S1). A mass interval of 79.7 Da between theoretical mass without modification and actual mass for fragment y9 was calculated, indicating that the site was phosphorylated.

### 2.3. Gene Ontology (GO) Analysis of Identified OC Proteins

The 628 proteins identified in LC-MS/MS analyses were functionally clustered by GO analysis with The Database for Annotation, Visualization and Integrated Discovery (DAVID) v6.7 by the National Institute of Allergy and Infectious Diseases (NIAID) as described [25]. 599 unique *Mus musculus* genes were recognized by DAVID and categorized into a wide spectrum of biological processes, molecular functions, and cellular components (Table S2). Duplicated and overlapped items in these three categories were merged and removed after validation.

**Figure 4:**
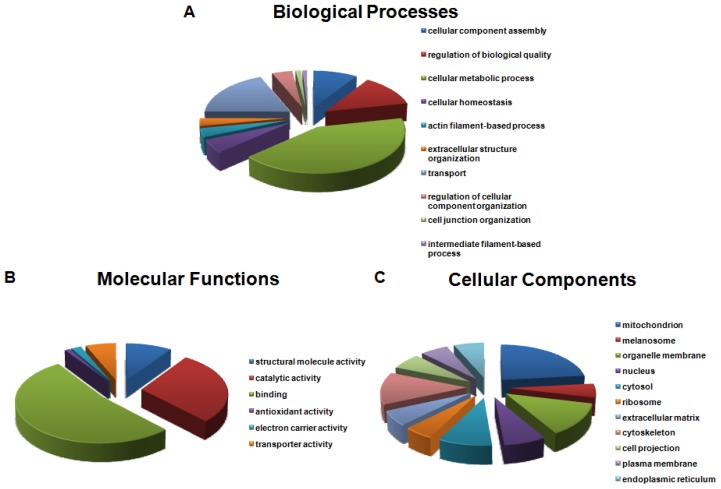
Gene Ontology (GO) analysis of the OC proteome with The Database for Annotation, Visualization and Integrated Discovery (DAVID) v6.7. Overrepresented categories with *p*-value < 0.05 were shown in each type. The area of a sector is proportional to the number of genes in the OC proteome annotated to the corresponding GO category. (**A**) Biological processes; (**B**) Molecular functions; (**C**) Cellular components.

Biological processes of proteins identified in the OC were plotted in Figure 4A. Twenty-seven were categorized as proteins involved in cellular homeostasis and comprised of 7 ion channels and ion binding proteins, including a cytoplasmic aconitase, Na^+^/K^+^-transporting ATPase α2 and α3, solute carrier family 12, sarcoplasmic/endoplasmic reticulum calcium ATPase 2, nucleobindin-2, and serotransferrin. Another six proteins belong to cell junction organization functioning in cell-cell adhesion, including catenin α-1, ponsin, laminin subunit γ-1, myelin protein P0 (MPZ), talin-1, and THY-1 membrane glycoprotein. Proteins identified in the OC were divided into six categories of varying molecular functions (Figure 4B). Approximately half of the proteins were associated with binding activity, including nucleotide, ribonucleotide, ATP, GTP, cytoskeletal protein, cofactor, actin, and coenzyme binding. In addition, 56 proteins fell within the categories of electron carrier activity and transporter activity. These proteins are encoded by genes representing ion transporters or channels, such as *Atp1a1*, *Atp1a2*, *Atp1a3*, *Atp5h*, *Slc25a12*, *Vdac1*, *Vdac2*, *and Vdac3*. Figure 4C illustrates cellular components of the identified proteins in the OC. A total of 31 proteins were expressed in the extracellular matrix, products of which are essential for transformation of the mechanical energy into electrical signals, and these included collagen α-1(XI) chain, α-tectorin (TECTA), and β-tectorin (TECTB).

## 3. Discussion

Strategies for the inner ear proteomic investigations were proposed in 2006 [11,26], and proteomic data were obtained from whole and subdissected inner ear tissues and also benefitting from development of micro-sample procurement protocols [11,15–21,27]. Proteomics of the stereocilia [28–31] and hair cell synaptic ribbons [32], in combination with transcriptomic and genomic approaches, have also yielded significant mechanistic insights to the functions of these structures at a molecular level. Technical advances in the proteomic analysis of small samples [23,33] have facilitated our investigation of the OC proteome. We were able to detect a total of 628 proteins from six combined LC-MS/MS analyses by OMSSA with 175/628 (27.8%) proteins identified by more than one unique peptide. Another advancement, illustrated in this study, was the identification of 28 modified proteins using the nanoLC-MS/MS based proteomics. PTMs mark a series of biological events that determine protein activity, interactions, turnover and localizations. The GO analysis of these 628 proteins suggested that a number of them had biological processes, molecular functions and cellular components that were relevant to the inner ear. While high throughput nanoLC-MS/MS based proteomic studies have been carried out on more complex biological samples with lengthy lists of proteins [34], this study represents our initial efforts in characterizing the OC using this methodology. As a shotgun proteomic approach, identification of these phosphorylated and acetylated proteins further verified the great potentials of our experimental protocols and our nanoLC-MS/MS platform in analyzing PTMs in minute amount of samples, especially without two-dimensional gel electrophoresis separation or affinity-based enrichment. Precisely how to interpret this PTM datasets and extract useful information is still problematic, but our efforts represent a significant step in exploiting this approach. In light of the complex anatomical structure, cellular diversity and physiological function of the OC proteins, the utilization of this highly specialized and unique tissue provides an excellent paradigm to test a small sample size for the detection and classification of a wide spectrum of proteins into cellular homeostasis, transport, and extracellular structure organization in biological process and electron carrier activity in molecular function.

Semi-quantitative analysis of the OC proteome shows its dynamic range is 10^4^ and detecting proteins with such a large NSAF range proved the sensitivity and reliability of our nanoLC-MS/MS platform in profiling complex protein samples. The most abundant protein detected was COCH. COCH is widely expressed in the OC and also represents one of the more abundant transcripts identified in cochlear cDNA libraries [35–37] as well as one of the most abundant proteins in the OC [35,38]. COCH is the major component of the OC extracellular matrix and is also deposited in neural channels and perivascular areas [35]. TECTA (230 kDa) was another abundant protein present in our OC preparation. TECTA, in addition to collagen, is a major protein present in the tectorial membrane [39–41]. TECTB was also identified but is less prevalent due to its lower concentration in the tectorial membrane and smaller molecular mass (32.5 kDa) [42,43]. Integrity of the extracellular matrices that comprise both the tectorial membrane and the basilar membrane is crucial for sustaining the function of the inner ear to fulfill mechanical stimulation [6]. Besides TECTA and TECTB, several other extracellular matrix proteins in the OC preparation were identified and included collagen α-1(II) chain, collagen α-1(XI) chain, and collagen α-2(XI) chain, encoded by *Col2a1*, *Col11a1*, and *Col11a2* genes, respectively. Otoancorin (OTOA) was also detected and is located at the interface between apical surface of the OC and the attachment site of tectorial membrane [44]. OTOA serves as a glycosylphosphatidylinositol linked membrane-bound protein. The tectorins and collagens are major elements in the acellular membranes and extracellular matrices of the cochlea.

Since hair cells are the neurosensory receptors of the inner ear, their proteomes represent a secondary constituent of the OC preparation that reflects the proportional abundance of this cell type. Thus, proteins unique to hair cells could determine the lower end of sensitivity of our detection. We were able to find the more abundant myosin isoforms, myosin VI (MYO6) and myosin IX (MYH9), which are unique to hair cells in OC. MYO6 is primarily concentrated at the cuticular plate of the inner and outer hair cells [45] with an essential role in maintaining the structural integrity of the stereocilia bundle [46]. An additional role was recently found in the association of MYO6 with synaptic ribbons of hair cells and is thought to play a role in neurotransmitter release [47]. MYH9 was also found, but has a wider expression pattern than being associated with the hair cells of the OC [48,49]. The expression of MYH9 is also abundantly present in the spiral limbus and spiral ligament of the cochlea [48,49]. In the hair cells MYH9 is localized to the stereocilia and cuticular plate, but it was also detected in the plasma membrane and mitochondria [50]. Other hair cell relevant myosin isoforms, such as myosin Ic (MYO1C), myosin IIIa (MYO3A), myosin VIIa (MYO7A) and myosin XV (MYO15A), were not detected. These myosins are associated with the stereocilia and the cuticular plate that represent the apical specialization of hair cells [22]. Actins are another major component of the stereocilia and the cuticular plate in the OC [51,52]. γ-actin, detected in the OC, was necessary to maintain the integrity of stereocilia [53]. Several other detected proteins, which are, in general, ubiquitously expressed, were otospiralin [54], Sparc [36] and F-box protein 2 (FBXO2, alias-organ of Corti protein 1 (OCP1)) [55–57]. Two other proteins, proteolipid protein 1 (PLP1) and MPZ, were found that have a restricted expression in supporting cells [58,59]. Many of the genes required for the unique structure and function of the auditory peripheral organ are restricted in their expression to the OC and were identified in our analysis that are associated with hearing loss (see Table 1).

Moreover, we compared the proteome of the OC obtained from our nanoLC-MS/MS analyses with previously published data of the OC and hair bundles for discovering the important molecules in the OC. Due to breakthroughs of microscale analysis resulting from refinement of inner ear tissue separation and purification, sensitivity and specificity of the analyses have seen significant progress [16]. A major advancement was the analysis of the proteome of hair bundles from chicken utricles [18]. By Shin *et al.* (2007) profiled 59 proteins of which we were able to detect 30 of these in our OC proteome dataset (Table S3). Currently, there is still limited data available for comparison of inner ear proteomic datasets. Other previous proteomic studies analyzed multiple inner ear tissues, which shared an identification of varieties of cytoskeletons, histones, and ion channels [20,27,32,60–62]. Microarray analyses have been performed within the OC, lateral wall and spiral ganglion of normal hearing CF-1 mice so as to determine the relative gene expression levels of each tissue [63]. Of the 29 more prevalent transcripts in the OC, only eight proteins (transcribed by *Ceacam16*, *Lect1*, *Otos*, *Epyc*, *Otoa*, *Skp1*, *Cndp2*, and *Cldn9*) were found in our protein dataset. Carcinoembryonic antigen-related cell adhesion molecule 16 (CEACAM16), an adhesion protein concentrating at the top of OHC stereocillia, interacts with TECTA and mutation of *CEACAM16* results in autosomal dominant nonsyndromic deafness (ADNSHL) at the DFNA4 locus [64]. FBXO2 binds to SKP1, forming the Skp1/Cullin1/FBox/Rbx1 (SCF) ubiquitin ligase complex, which targets select proteins for degradation by the 26S proteasome [65]. Hair cell and spiral ganglion neurodegeneration were found in *Fbxo2*^−/−^ mice with decreased *Skp1* expression in the cochlea, resulting in progressive hearing loss at 2 months of age [66]. Another protein identified was Claudin-9 (CLDN9). CLDN9 is a tight junction-associated protein ubiquitously expressed in the inner ear, and was shown to provide an ion barrier that prevents the leakage of K^+^ from the K^+^-rich endolymph into the basolateral fluid of hair cells and the subsequent loss of OHCs [67]. Our data also consist of a number of proteins involved in glycotic pathway which was the dominant energy source in the stereocillia, including creatine kinase B, which is crucial for maintaining ATP levels in hair cells and satisfying the most significant consumption of ATP by the plasma-membrane Ca^2+^-ATPase isoform 2 (PMCA2) to remove Ca^2+^ entering stereocillia [18]. With shared calretinin, proteins involved in Ca^2+^ binding were also detected in the OC, including calnexin and parvalbumin α.

Quite a number of significant proteins have been identified in our proteomic analysis of the mouse OC, but more in-depth studies are needed to provide a comprehensive view of the molecules involved in organ development and auditory function as well as hearing impairment in the OC. There are a number of genes in the inner ear that are exclusively expressed in hair cells and can vary in their relative abundance. In general, hair cell-specific myosin proteins should provide a range in concentration with MYO6 and MHC9 being generally more abundant than MYO7A and MYO15, which have lower concentrations. These hair cell-specific proteins can be used as a parameter to determine the scope and range of the proteomic analyses. Besides these myosin proteins, another protein which is highly expressed only in outer hair cells is prestin (SLC26A5), making it a good biomarker for proteomic studies of neurosensory cells. Other low abundant hair cell relevant proteins include several transcription factors, which are the atonal homolog 1 (ATOH1) protein, POU domain 3c (POU4F3) protein and zinc finger growth factor independent 1 (GFI1) protein. Use of a supporting cell-associated transcription factor, SOX2, could also be used to determine the sensitivity of the proteomic analyses. These are only some of the molecules that are important and relevant to the different cell types within the OC. Ion transporters and channels in the inner ear, especially K^+^ channels, are essential for ion homeostasis and hair cell polarization. Two prevalent gap junction proteins of the inner ear are connexin 26 (gap junction β-2-GJB2) and connexin 30 (GJB6). We were able to identify the GJB6 peptide in the OC samples. Gap junction channels are known to be responsible for recirculation of K^+^ ions during hair cell excitation [68]. Besides these, there are other genes encoding tight junction proteins, synaptic transmission proteins and ion transport pathways between the cells of the cochlea. These genes are essential for function of the peripheral auditory system. With the recent breakthroughs in nanoLC-MS/MS, the current proteomic analysis should permit an expanded elucidation of the OC proteome in order to fully characterize the proteins, their modifications and quantities required for maintenance and function of the OC. In addition, as the technologies advance, separate profiling of inner and outer hair cells could manifest their differential but intimately related functions in the OC. Therefore, our proteomic analysis of the OC in the inner ear identified a number of proteins of structural and functional significance and bridges the gap between genes, transcripts and proteins in the OC. Future proteomic study of OC and more particularly the unique cell types in the OC can further contribute to the discovery of novel biomarkers for clinical diagnosis and the development of *in vivo* gene therapy for hearing loss.

## 4. Experimental Section

### 4.1. OC Isolation

Animal handling complied with protocols approved by the Creighton University Institutional Animal Care and Use Committee (IACUC # 0730). Five normal hearing FVB/N male mice (4–5 weeks) (Harlan, Indianapolis, IA, Indiana) were euthanized and tympanic bullae were dissected and immediately placed in cold Minimum Essential Medium (Invitrogen Corp., Carlsbad, CA, USA) for further delicate dissection. After removal of the temporal bone, the cochleae were dissected out at 4 °C. The cochlear wall was trimmed away from the oval window toward the apex. Then following removal of the stria vascularis, the OCs were carefully unwrapped from the osseous spiral lamina from the modiolus toward the apex and then retrieving the OC in an apical to basal progression. After visual inspection of the integrity, the samples were stored at −20 °C.

### 4.2. In-Solution Trypsin Digestion

OCs were rinsed and reconstituted in 100 μL of lysis buffer (100 mM ammonium bicarbonate (SigmaAldrich, St. Louis, MO, USA), pH 8.4). Cell lysates were centrifuged at 13,000 rpm for 20 min. The supernatants were saved for trypsin digestion. RapiGest™ SF (Waters, Milford, MA, USA) powder was dissolved in 100 mM ammonium bicarbonate (pH 8.4) to obtain 1% RapiGest™ SF solution (*w*/*v*). Protein pellets were suspended in 1% RapiGest™ SF solution at a final concentration of 0.1%. Disulfide bonds were reduced with 5 mM DTT (SigmaAldrich, St. Louis, MO, USA) at 60 °C for 30 min. The sample was cooled to room temperature (RT) and incubated with 15 mM iodoacetamide (SigmaAldrich, St. Louis, MO, USA) in the dark at RT for 30 min. Protein concentration was determined by Coomassie Brilliant Blue protein assay (Thermo Fisher Scientific, Rockford, IL, USA). Then sequencing grade trypsin (Promega, Madison, WI, USA) was added to the protein solution at a ratio of 1:5 (*w*/*w*). The sample was incubated at 37 °C overnight. pH of the sample was reduced to 3.0 with 2% trifluoroacetic acid (TFA) (SigmaAldrich, St. Louis, MO, USA) and the sample was incubated at 37 °C for 60 min. The sample was promptly frozen in liquid nitrogen to further precipitate surfactant. The sample was centrifuged at 16,000 rpm for 5 min and the supernatant was transferred to a clean 1.5 mL microcentrifuge tube. After elevation of pH to ~7.0 with dilute ammonium bicarbonate, peptide concentration was measured by bicinchoninic acid (BCA) protein assay (Thermo Fisher Scientific, Rockford, IL, USA). The digests were desalted using reversed-phase Sep-Pak cartridges (Waters, Milford, MA, USA) and dried in a vacuum centrifuge. The digested sample was stored at −80 °C until LC-MS/MS analysis.

### 4.3. LC-MS/MS Analysis

The desalted and purified peptides were analyzed by online nanoLC-MS/MS. Liquid chromatography was performed using an in-house built metal-free nanoLC system which was constructed by our lab in terms of the initial introduction by Zhao & Ding, *et al*. [33]. The columns were prepared by slurry packing 3 μm C18 bonded particles (Michrom Bioresources, Auburn, CA, USA) into a 50 μm i.d. × 40 cm PicoFritTM column with an integral 5 μm emitter (New Objective, Woburn, MA, USA). For LC-MS/MS analysis, 183 μg digested peptides were dissolved in 1 mL of (95% (*v*/*v*)) double distilled H_2_O, 5% (*v*/*v*) acetonitrile (Thermo Fisher Scientific, Rockford, IL, USA), and 0.1% (*v*/*v*) formic acid (Thermo Fisher Scientific, Rockford, IL, USA). 5 μL of sample solution containing less than 1 μg peptide digests was loaded onto the pre-column which was built in our nanoLC system. The mobile phase A consisted of 0.1 M acetic acid in double distilled water. The nanoLC system was operated under a constant-pressure mode for gradient generation, which resulted in gradients with exponential profiles [69]. Peptide separation was achieved by increasing the mobile phase from 0 to 60% (0.1 M acetic acid, 80% (*v*/*v*) acetonitrile, 20% (*v*/*v*) double distilled H_2_O) for 400 min. The nanoLC system was interfaced with a LTQ-Orbitrap XL mass spectrometer equipped with a nanoelectrospray ion source (Thermo Scientific, Waltham, MA, USA). During identification of the eluting peptides by the mass spectrometer, a full MS scan was followed by 10 MS/MS scans in the data-dependent mode with automatic switch. Full MS spectra (*m*/*z* from 300 to 2000) were acquired in the Orbitrap with resolution of 60,000. Top 10 precursor ions (signal intensity dependent) were sequentially isolated and then simultaneously fragmented in the linear ion trap by collision-induced dissociation. The heated capillary was maintained at 175 °C, while the spray voltage was kept at 2.2 kV.

### 4.4. Protein Identification

Raw MS/MS files were processed with DeconMSn (version 2.2.2.2, Pacific Northwest National Laboratory (PNL)), DtaRefinery (version 1.1, PNL) and DtaTextToMGF (version 1.2.3952, PNL) to produce mgf files. Sequence assignment was performed using OMSSA MS/MS search engine [70] (version 2.1.9, National Center for Biotechnology Information (NCBI)) against the mouse International Protein Index (IPI) protein sequence database (Version 3.52) [71] containing the normal IPI mouse proteins, commonly observed contaminants, and the reverse sequences of all proteins, with precursor mass tolerance of ±0.02 Da and product ion tolerance of ±0.50 Da. A maximum of two miscleavages were allowed. In the database searching, carbamydomethylation of cysteine was set as fixed modification, and acetylation of protein *N*-terminal and lysine, phosphorylation of serine, threonine, and tyrosine, and oxidation of methionine were set as variable modifications. The OMSSA output csv files were combined for FDR calculation. To ensure our identification results, we applied a 1% FDR cutoff sequentially at the spectrum level, the peptide level, and the protein level using the decoy database approach as previously described with modifications [72,73]. First, all identified peptide-spectrum matching (PSM) results were sorted by the quality of peptide identification (QPI) value calculated as −log10 (OMSSA *E*-value) and 1% FDR cutoff was applied to remove the low-confident PSM results. Second, the QPI value of a unique peptide was calculated as the sum of the QPI values of all PSM results that were identified as this peptide. Then the FDR cutoff of 1% was applied at the unique peptide level. Third, 1% FDR cutoff was applied at the protein level based on the QPI value of a protein, which was calculated as the sum of the QPI values of all unique peptides that were derived from the protein.

### 4.5. Semi-Quantitative and GO Analyses

Semi-quantitative analysis of identified proteins was performed by normalizing spectra count with NSAF [3,74]. GO analysis [75] was performed by DAVID v6.7 [25].

## 5. Conclusions

From our nanoLC-MS/MS based proteomic study, 628 proteins were identified in six replicates of single LC-MS/MS analysis from the OC of normal hearing FVB/N male mice by OMSSA. Dynamic range of the OC proteome achieved in this study is 10^4^. Cochlin was found to be the most abundant protein present in the OC. A total of 28 modified proteins were identified. Eleven hearing impairment related proteins, including cochlin, myosin VI, and myosin IX, were identified in the mouse OC sample. Furthermore, GO analyses revealed that about 10% of identified proteins were classified as electron carriers and transporters and 5% were expressed in extracellular matrix, which maintain the integrity of the structure and function of the OC. These findings can enhance our understanding of the OC proteome from normal hearing animals and provide the basis of future proteomic studies aiming at identification of protein markers for early clinical diagnosis and novel drug targets to restore hearing.

## Figures and Tables

**Table 1:** Identification of hearing impairment related proteins in the mouse organ of Corti (OC) sample.

Protein Name	IPI ID^a^	Gene Name	Locus^b^
γ-actin	IPI00648420	*Actg1*	DFNA20
Cochlin	IPI00127100	*Coch*	DFNA9
Collagen α-1(XI) chain	IPI00230065 IPI00400048	*Col11a1*	STL2
Collagen α-2(XI) chain	IPI00283629	*Col11a2*	DFNA13 DFNB53 STL3
Collagen α-1(II) chain	IPI00828467	*Col2a1*	STL1
Gap junction β-6 protein	IPI00111902	*Gjb6*	DFNA3B DFNB1
Myosin IX	IPI00123181	*Myh9*	DFNA17
Myosin VI	IPI00462752 IPI00776187	*Myo6*	DFNA22 DFNB37
Otoancorin	IPI00265452	*Otoa*	DFNB22
α-tectorin	IPI00114338 IPI00415299	*Tecta*	DFNA12
Carcinoembryonic antigen-related cell adhesion molecule 16	IPI00356708	*Ceacam16*	DFNA4

a International Protein Index;

b Gene loci were obtained from The Hereditary Hearing loss Homepage [24].
